# Dysexecutive syndrome and cerebrovascular disease in non-amnestic mild cognitive impairment: A systematic review of the literature

**DOI:** 10.1590/S1980-57642012DN06030006

**Published:** 2012

**Authors:** Felipe Kenji Sudo, Carlos Eduardo Oliveira Alves, Gilberto Sousa Alves, Letice Ericeira-Valente, Chan Tiel, Denise Madeira Moreira, Jerson Laks, Eliasz Engelhardt

**Affiliations:** 1 Instituto de Psiquiatria, Center for Alzheimer’s Disease (CDA/IPUB), Universidade Federal do Rio de Janeiro, Rio de Janeiro RJ, Brazil;; 2 Instituto de Neurologia Deolindo Couto (INDC), Universidade Federal do Rio de Janeiro, Rio de Janeiro RJ, Brazil;; 3 Cognitive and Behavioral Neurology Unit, INDC-CDA/IPUB, Universidade Federal do Rio de Janeiro, Rio de Janeiro RJ, Brazil;; 4 Universidade do Estado do Rio de Janeiro;; 5 Hospital Pró-Cardíaco, Rio de Janeiro, Rio de Janeiro RJ, Brazil.

**Keywords:** mild cognitive impairment, vascular dementia, executive function, neuropsychology, neuroimaging, cerebrovascular disease

## Abstract

**Objective:**

Non-amnestic dysexecutive Vascular Mild Cognitive Impairment (VaMCI) may represent preclinical Vascular Dementia (VaD). The aim of this study was to summarize the clinical, neuropsychological and neuroimaging aspects of VaMCI; and to assess its patterns of progression to dementia.

**Methods:**

Searches were made in the ISI Web of Knowledge, PubMed and Lilacs databases, using the terms "mild cognitive impairment" and "executive function". Altogether, 944 articles were retrieved.

**Results:**

VaMCI cases had poorer performances on fronto-executive tasks, a higher prevalence of stroke, presence of periventricular and profound white matter hyperintensities on MRI images, as well as more extrapyramidal signs and behavioral symptoms. Executive dysfunction might be associated with disconnection of fronto-parietal-subcortical circuits. Progression to dementia was associated with baseline deficits in executive function, in simple sustained attention and language, and large periventricular WMH.

**Discussion:**

VaMCI develops with impairment in non-memory domains and subcortical white matter changes on MRI images, which are consistent with clinical and neuroimaging findings in VaD.

## Abstract (pt)

**Objetivo:**

Comprometimento Cognitivo Leve Vascular (CCLV) não-amnésico disexecutivo pode representar a fase pré-clínica da Demência Vascular (DV). Este estudo tem por objetivo descrever os aspectos clínicos, cognitivos e de neuroimagem do CCLV, além de avaliar padrões de progressão para demência.

**Métodos:**

Foram realizadas buscas nas bases de dados ISI Web of Knowledge, PubMed e Lilacs, usando os termos "comprometimento cognitivo leve" e "função executiva". Ao todo, 944 artigos foram obtidos.

**Resultados:**

Pacientes com CCLV apresentaram piores desempenhos em tarefas fronto-executivas, histórico de acidentes vasculares encefálicos, presença de hiperintensidades da substância branca periventriculares e profundas, e mais, sinais extrapiramidais e sintomas comportamentais. A disfunção executiva parece se relacionar à desconexão de circuitos fronto-parieto-subcorticais. A progressão para demência relacionou-se a déficits em linguagem, função executiva e atenção sustentada à primeira avaliação, e a hiperintensidades na substância branca periventricular extensas.

**Discussão:**

O CCLV cursa com comprometimento cognitivo em domínios não-memória e alterações da substância branca subcortical à imagem por RM alinhadas com os achados característicos da DV.

## INTRODUCTION

Mild Cognitive Impairment (MCI) refers to an intermediate stage of cognitive decline between normal aging and dementia. Although early studies focused on the association between amnestic-subtype MCI (aMCI) and Alzheimer's disease (AD),^1,2^ empirically-based population studies have suggested that prodromal phases of both neurodegenerative and vascular dementias might pass through non-amnestic presentations of MCI.^3,4^ In 2003, an international working group met in Stockholm and expanded the concept of MCI to encompass pre-dementia syndromes related to other outcomes.^5^ Petersen and colleagues proposed a diagnostic algorithm for identifying MCI patients and predicting possible etiologies and progression patterns, according to subtypes. This classification system differentiated four subgroups of MCI: amnestic (single and multiple domains) and non-amnestic (single and multiple domains). Non-amnestic single-domain MCI could represent preclinical Fronto-temporal Lobar Degeneration, whereas non-amnestic multidomain MCI might be at-risk for progression to Vascular Dementia (VaD).^6^ In fact, some recent studies using clustering techniques have demonstrated different subtypes of MCI according to impaired cognitive domain. Delano-Wood et al. reported evidence for an amnestic group, an amnestic/language group, a dysexecutive/information processing speed group, and a mixed multidomain group.^7^ Similarly, Libon et al. found evidence for an amnestic group and a dysexecutive group.^8^ The American Psychiatric Association included, in a preliminary draft for the upcoming 5^th^ edition of the Diagnostic and Statistical Manual of Mental Disorders (DSM-5), the diagnostic criteria for Minor Cognitive Disorder, which would be a predementia condition analogous to MCI related to different etiologies such as AD, Vascular Disease, Fronto-temporal Lobar Degeneration, Traumatic Brain Injury, Lewy Body Disease, Parkinson's Disease, HIV infection, Substance Abuse, Huntington's Disease and Prion Disease.^9^

Non-amnestic MCI (non-aMCI) corresponds to individuals with predominant impairment in one or multiple non-memory domains that is not of sufficient magnitude to meet criteria for dementia. Prevalence estimates for non-amnestic MCI range from 17 to 38% of all MCI subjects.^3,10^ Single-domain non-amnestic MCI individuals seem to be less likely to convert to dementia than aMCI, but a study suggested that they might have higher rates of death over 5 years.^4^ Dysexecutive mild cognitive impairment (dMCI) can be defined as single-domain MCI with scores at or below the 10^th^ percentile of control performance on at least one executive function task, and scores within one standard deviation of normal means in memory assessment.^11^ This might be a relatively common condition with a mean prevalence of 16.77% among MCI individuals.^8,12,13^

There is growing evidence that MCI cases with impairment in executive function (EF) may present cerebrovascular disease (CVD). This group might exhibit greater white-matter lesion (WML) volumes in comparison to groups with MCI due to Alzheimer Disease (MCI-AD).^7^ A possible neuroanatomic mechanism driving dysexecutive syndrome might be the interruption of reciprocal and intimate connections between prefrontal cortex and the dorsal medial nucleus of the thalamus; in dMCI, it is possible that downwardly projecting pathways from prefrontal cortex towards the thalamus might be compromised, limiting the capacity of the prefrontal cortex to engage in its superordinate executive functions.^12^ Likewise, a previous study from this group showed that, compared to NCs, dMCI patients had higher severity of periventricular and profound WML as well as more depressive symptoms and lower scores on CLOX, Trail-Making Test, praxis, abstraction and CAMCOG's global score.^14^ Thus, individuals with MCI suffering from dysexecutive syndrome might also be associated to high-risk for progression to VaD.^15^

Our objectives in this systematic review were as follows: to describe clinical, neuropsychological and neuroimaging aspects of vascular-related non-amnestic dysexecutive MCI patients, and to assess patterns of progression to dementia of non-amnestic VaMCI.

## METHODS

A review of the literature was performed through searches in the electronic databases PubMed (http://www.ncbi.nlm.nih.gov/pubmed/), Institute for Scientific Information Web of Knowledge (http://www.isiknowledge.com) and LILACS (http://lilacs.bvsalud.org/), using the terms "mild cognitive impairment" and "executive function". We also hand-searched articles cited in the selected papers, so that publications missed by the electronic research could be added.

Inclusion criteria were as follows: original articles written in English, Spanish, Portuguese or French; studies focusing on vascular-related non-amnestic dysexecutive subtypes of MCI, and papers that evaluated neuropsychological assessment of executive function in Vascular MCI (VaMCI). Posters, reviews and case-reports were excluded from this review, as were papers published prior to Petersen's 2004 revised criteria for diagnosis of MCI.^6^ Studies using predementia clinical constructs other than MCI were also excluded from this study.

Studies retrieved by the electronic searches were analyzed by two independent reviewers (F.K.S. and E.E.), who selected all articles relevant to this review according to the inclusion criteria.

## RESULTS

**Figure 1:**
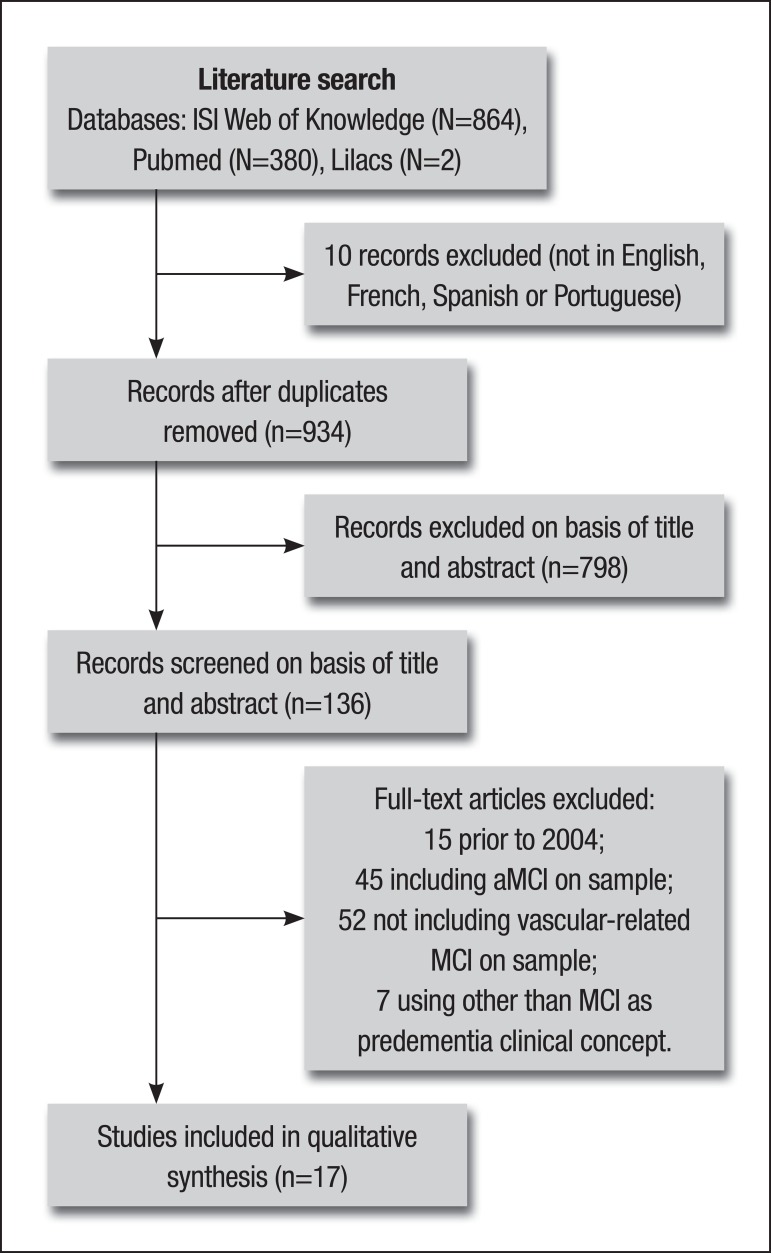
Flow diagram describing the systematic review of the literature on dysexecutive syndrome in vascular -related non -amnestic MCI.

Of the initially retrieved 944 articles, 17 met our criteria. A flow chart was designed to summarize the different phases of the research, as recommended by The PRISMA Work Group for reporting systematic reviews and meta-analyses^16^ (Figure 1). Characteristics of the selected studies are depicted in Tables 1, 2 and 3. Only 6 papers focused on single-domain dysexecutive MCI (dMCI), but in all of these the samples comprised patients with no significant vascular lesions on brain MRI, since authors had excluded patients who presented history of stroke^7,8,12^ and a Longstreth grade above 4 on MRI imaging.^11,13,17^ These articles were excluded from our study because of this reason. Papers focusing on dysexecutive vascular-related non-amnestic MCI were included in this study. Only one of the articles was a population-based study and the remaining 16 drew on samples from tertiary memory disorder clinics and research database. Five were longitudinal studies.

**Table 1:** Characteristics of studies on clinical and neuropsychological aspects of non-amnestic VaMCI included in this review.

Author/year	Design	Setting	MCI (n)	Findings
Galluzzi et al., 2005	CS	TC	43	Extrapyramidal sign scale, letter fluency, items “irritability” and urinary dependence on NPI, and digit span forward discriminated subcortical VaMCI from degenerative MCI.
Zanetti et al., 2006	L	TC	65	Dysexecutive syndrome, vascular comorbidity, vascular lesions on tomography brain scan, higher prevalence of extra pyramidal features, mood disorders, and behavioral symptoms were found in mdMCI in comparison to aMCI.
Gainotti et al., 2008	CS	TC	77	EF was a weak cognitive marker of CVD in MCI, whereas episodic memory was strongly associated to MCI-AD.
Knopman et al., 2009	CS	PB	329	History of stroke and impairment in non-memory cognition was associated to non-aMCI . Presence of APOE4 was associated to aMCI.
Zhou and Jia, 2009	CS	TC	86	VaMCI were mainly mdMCI, MCI-AD presented memory and EF impairments. VaMCI presented better memory performances and worse processing speed compared to MCI-AD.
Teng and al., 2010	CS	TC	1108	IADL deficits were greater in amnestic than non-aMCI groups, but within these subgroups, did not differ between those with single or multiple domains of cognitive impairment. IADL deficits were present in both aMCI and non-aMCI but not related to the number of impaired cognitive domains.
Sudo et al., 2010	CS	TC	20	CAMCOG's Abstract thinking subtest and CAMCOG's total score discriminated VaMCI from NC.
Saunders and Summers, 2011	L	TC	81	aMCI and non-aMCI display a stable pattern of deficits to attention, working memory, and executive function. The decline in simple sustained attention in aMCI and non-aMCI groups and in divided attention in aMCI may be early indicators of possible transition to dementia from MCI.
Hanfelt et al., 2011	CS	TC	1655	MCI subgroups with functional and neuropsychiatric features were at least 3.8 times more likely than the least impaired MCI group to have a Rosen-Hachinski score of 4 or greater, an indicator of probable CVD.
Ambron et al., 2012	CS	TC	154	The frequency of close-in behavior was significantly higher in multidomain non-aMCI than in multidomain aMCI, suggesting that CIB is not associated with specific memory impairment. Patients with closing-in behavior were slightly but significantly more impaired on executive function tasks.

L: longitudinal; CS: cross-sectional; PB: population-based; TC: tertiary center; MCI-AD: MCI due to AD; VaMCI: vascular MCI; non-aMCI: non-amnestic MCI; aMCI: amnestic MCI; mdMCI: multiple domain MCI; sdMCI: single-domain MCI; IADLs: instrumental activities of daily living.

**Table 2:** Characteristics of studies on neuroimaging aspects of non-amnestic VaMCI included in this review.

Author/year	Design	Setting	MCI (n)	Findings
Bombois et al., 2007	CS	TC	170	Prevalence of SH was high in MCI, irrespective of the subtype. Executive dysfunction was independently associated with SH, WML and PVH.
Shim et al., 2008	CS	TC	40	MCI showed decreased FA values and increased MD compared to NC. VaMCI showed greater FA decrease than non-VaMCI and NC. VaMCI showed greater EF impairments than non-VaMCI and NC.
Grambaite et al., 2011	CS	TC	23	Increased white-matter tract radial and mean diffusivity on DTI in frontal and cingulate regions and cortical thinning in caudal middle frontal region were both associated with executive dysfunction in MCI.
Jacobs et al., 2012	L	TC	337	WMH in the frontal-parietal and in the frontal-parietal-subcortical network were associated with decline in executive functioning. However, the frontal-subcortical network was not associated with change in executive functioning.

L: longitudinal; CS: cross-sectional; TC: tertiary center; VaMCI: Vascular MCI; SH: subcortical hyperintensities; WML: white-matter lesions; PVH: periventricular hyperintensities; FA: fractional anisotropy.

**Table 3:** Characteristics of studies on course of non-amnestic VaMCI included in this review.

Author/year	Design	Setting	MCI (n)	Findings
Zanetti et al., 2006	L	TC	65	Within 3 years, 31% of MCI progressed to dementia. All patients who evolved to AD had been classified as aMCI and all patients who progressed to VaD had been identified as mdMCI.
Debette et al., 2007	L	TC	170	Patients who declined over 3.8-year follow-up in MMSE scores had larger amounts of PVH and WMH, compared to those who did not decline. Decline in Mattis Dementia Rating Scale was related only to PVH. Larger PVH was predictive of decline in EF. The association between PVH and cognitive decline was irrespective of MCI subtype.
Sachdev et al., 2009	L	TC	45	Post-stroke VaMCI showed greater decline in logical memory, more vascular risk-factors and more WML than NC over 3 years. Neither MRI volumetric measurements nor cerebrovascular events predicted decline.
Saunders and Summers, 2011	L	TC	81	aMCI and non-aMCI displayed a stable pattern of deficits in attention, working memory, and executive function. The decline in simple sustained attention in aMCI and non-aMCI groups and in divided attention in aMCI may be early indicators of possible transition to dementia from MCI.

L: longitudinal; TC: tertiary center; VaMCI: vascular MCI; non-aMCI: non-amnestic MCI; aMCI: amnestic MCI; mdMCI: multiple domain MCI; PVH: periventricular hyperintensities, WMH= profound white-matter hyperintensities.

**Clinical features and assessment of fronto-executive functions.** Individuals with VaMCI had poorer performances on frontal neuropsychological tests compared to neurodegenerative MCI in most studies.^18^ Among the tests used to evaluate fronto-executive functions, the Stroop Test^18^ and letter fluency^19^ discriminated VaMCI from MCI-AD. MCI-AD had better performance in processing speed compared to VaMCI, whereas the former presented better results on memory tests compared to the degenerative subtype.^18^ However, one study indicated that executive dysfunction might not be as consistently associated to VaMCI as episodic memory impairment is related to MCI-AD.^20^ Prior stroke in MCI subjects was independently associated with lower cognitive performances across all non-memory domains in comparison to NC, and scores on the TMT B and Digit Symbol Substitution were more strongly associated with history of stroke.^21^ The Abstract Thinking subtest from the CAMCOG distinguished VaMCI from NC in one study.^22^

A high prevalence of arterial hypertension, severity of WML on neuroimaging and presence of extrapyramidal motor signs were identified in non-amnestic mdMCI patients. Planning, problem solving and working memory were particularly impaired in mdMCI in comparison to sdMCI.^23^ Moreover, history of stroke was associated with a higher risk of developing non-aMCI than aMCI.^18,21^ A large multicenter study reported that MCI patients with neuropsychiatric and functional deficits might be more likely to have CVD than MCI with only cognitive impairment.^24^ Closing-in behavior, which is a tendency in figure copying to draw very close to or on top of the model, was identified in non-amnestic mdMCI and was associated with executive dysfunction.^25^

Ability to perform instrumental activities of daily living showed no association with number of impaired cognitive domains in one study.^26^ In spite of presenting poorer performances on EF tasks and progressing with more behavioral symptoms,^27^ non-aMCI may suffer less functional deficits than aMCI, according to one study.^26^

**Neuroimaging.** Age was independently and positively associated with severity of white-matter lesions on structural brain MRI imaging. In this same study, prevalence of subcortical white matter hyperintensities (WMH) corresponded to 92% of the sample of MCI individuals. Periventricular and lobar white matter, especially in frontal lobe, were the most predominant locations of subcortical WMH although their presence was neither associated with global cognitive performance (MMSE and CDR) nor with MCI subtype. Nevertheless, severity of periventricular and profound WMH might specifically contribute to deficits in EF.^28^

Recent data reported that executive dysfunction might be associated with location of WMH. Lesions in fronto-parietal and fronto-parietal-subcortical networks were associated with decline in EF among MCI patients. WMH in fronto-subcortical tracts did not significantly contribute to impairment in EF in one study.^29^ Studies using diffusion tensor imaging (DTI) techniques with quantitative fractional anisotropy (FA) corroborated the importance of parietal-subcortical connections in dysexecutive syndromes. Quantitative DTI-FA mean values were lower in parietal regions and centrum semiovale in VaMCI, compared to both NC and non-vascular MCI. These findings were related to impairments in visuospatial and executive functions.^30^ On the other hand, disconnection of networks associated to frontal and temporal lobes might also be implicated in the pathophysiology of EF impairment in VaMCI. According to Grambaite et al., MCI patients with attentional and executive impairments might present, on DTI, increased ratio of white matter radial diffusivity/ mean diffusivity in frontal, cingulate and entorhinal regions, suggestive of regional demyelination and axonal atrophy. White-matter tract degeneration in frontal and cingulate regions, and cortical thinning in caudal middle frontal region, showed association with executive dysfunction.^31^

**Course and progression to dementia.** Among patients classified as mdMCI, 26% progressed to VaD in a 3-year follow-up, supporting a possible vascular etiology of this condition.^37^ Sachdev et al. identified a rate of conversion to dementia of 8% per year among patients with VaMCI.^32^ The main predictors of cognitive decline and progression from VaMCI to dementia listed in the studies were as follows: impaired EF and language at baseline,^32^ large amounts of periventricular WMH (but not profound WMH)^33^ and decline in simple sustained attention.^34^

Notably, some contradictory findings have been disclosed. Sachdev et al. reported that impaired cognitive performance at baseline might be a stronger predictor of progression to dementia than MRI measurements or intervals of cerebrovascular events.^32^ Additionally, large amounts of periventricular WMH were predictive of a more rapid decline in EF, especially in MCI patients with executive dysfunction at baseline according to Debette et al.^33^ Moreover, impairments in divided and sustained attention might be observed in the course of both non-aMCI and aMCI, but decline in simple sustained attention might be specifically related to risk for transition to dementia from non-aMCI.^34^

## DISCUSSION

Consistent with our expectations, there is mounting evidence linking CVD with non-amnestic multidomain MCI. Subcortical WMH might be a very common finding, occurring in 92% of a sample of MCI patients.^28^ Accordingly, a history of stroke and presence of periventricular and profound WMH on brain MRI images were associated with impairments in non-memory cognitive domains, especially EF in MCI individuals.^21^ History of hypertension,^23^ severity of WML,^23^ prevalence of behavioral symptoms,^24^ extrapyramidal signs,^23^ and closing-in behavior^25^ number among clinical and neuroimaging aspects observed in non-amnestic mdMCI, which is coherent with the putative role of CVD implicated in the etiology of cognitive impairment in this group.

Fronto-parietal-subcortical network disconnection is deemed to be the most important underlying substrate of dysexecutive syndrome in VaMCI.^29,30^ Interruption of white matter fibers in frontal lobe connections, as detected by DTI-FA techniques, has been reported as a possible neuropathological mechanism for dysexecutive syndrome in patients with Binswanger Disease.^35,36^ Likewise, studies using DTI-FA corroborated the importance of parietal-subcortical connections in dysexecutive syndromes.^29,30^ Reduction of anisotropy was identified in parietal regions and centrum semiovale in VaMCI, which was related to executive dysfunction. In addition, WMH in parietal lobes negatively impacts glucose metabolism in frontal lobes in individuals with CDR=0 or 0.5, which might contribute to impairment in executive function.^37^

Previous studies containing factor analysis of putative EF measures identified multiple dimensions of cognitive processing, such as abstract thinking, cognitive flexibility, working memory and response inhibition to distractors. Due to this broad range of functions reflecting the complexity of prefrontal cortical-subcortical circuits, no single neuropsychological test has been identified as a "gold standard" measure for EF. Therefore, in order to avoid under-evaluation of its components, clinical assessment of EF must include a comprehensive set of tasks, as opposed to a single measure.^38,39^ The Wisconsin Sorting Card Test (WSCT),^40^ Stroop Color/Word Interference Test,^41^ Iowa Gambling Test,^42^ EXIT-25,^43^ CLOX,^44^ Verbal Fluency,^45^ Trail-Making Test (TMT) forms A and B^46^ and Porteus' mazes test^47^ are among the most used instruments to assess EF in studies. In this review, neuropsychological tests that proved useful to distinguish between VaMCI and MCI-AD were the Stroop test^18^ and letter fluency,^19^ while the Abstract Thinking subtest in CAMCOG distinguished VaMCI from NC.^22^

Baseline predictors of progression for patients with VaMCI were impairments in EF and language,^32^ large periventricular WMH^33^ and decline in simple sustained attention.^34^ Rate of progression to dementia was approximately 8% per year in VaMCI individuals.^27,32^ Coherently, a previous study prospectively assessing patients with Vascular Cognitive Impairment No-Dementia, a clinical construct analogous to VaMCI, identified a rate of 46% of conversion to dementia after 5 years of follow-up.^48^

To our knowledge, the present study is the first to review the main clinical, cognitive and neuroimaging features of dysexecutive non-amnestic VaMCI. Several limitations of this study should be acknowledged. Firstly, no data were available in the literature concerning vascular single-domain dMCI. One previous study conducted by Eppig et al. (2012) analyzed EF performances in dMCI as a function of time and revealed a decline in Mental Control tests and letter fluency, in comparison to aMCI and normal controls.^12^ Moreover, dMCI patients showed overall cognitive performance consistent with cognitive deficits seen in dementia due to subcortical pathology, as shown by intact anterograde memory and impaired EF tasks.^12^ Further studies are needed to determine the influence of WML on cognitive function in vascular dMCI patients. Secondly, most of the studies included in this review used convenience samples from tertiary hospitals or memory clinics. Large prospective longitudinal studies, using population-based settings are needed. Such epidemiological studies of vascular-related dysexecutive MCI would advance our understanding of the role of CVD in early stages of cognitive impairment, its predictive value for future conversion to dementia and highlight which clinical and cognitive aspects can help differentiate VaMCI among neurodegenerative disorders.

In summary, the studies discussed in this review suggested that cognitive testing, including EF evaluation and assessment of severity of WMH, might be important both as diagnostic tools and as predictors of conversion to dementia in early stages of Vascular Cognitive Impairment.
